# An In Silico Investigation of the Molecular Interactions between Volatile Anesthetics and Actin

**DOI:** 10.3390/ph17010037

**Published:** 2023-12-26

**Authors:** Barbara Truglia, Nicola Carbone, Ibrahim Ghadre, Sara Vallero, Marinella Zito, Eric Adriano Zizzi, Marco Agostino Deriu, J. A. Tuszynski

**Affiliations:** 1 DIMEAS, Politecnico di Torino, 10129 Turin, Italy; 2 Department of Data Science and Engineering, The Silesian University of Technology, 44-100 Gliwice, Poland; 3 Department of Physics, University of Alberta, Edmonton, AB T6G 2E1, Canada

**Keywords:** anesthesia, actins, tubulin, cytoskeleton, molecular docking simulation

## Abstract

Volatile anesthetics (VAs) are medicinal chemistry compounds commonly used to enable surgical procedures for patients who undergo painful treatments and can be partially or fully sedated, remaining in an unconscious state during the operation. The specific molecular mechanism of anesthesia is still an open issue, but scientific evidence supports the hypothesis of the involvement of both putative hydrophobic cavities in membrane receptors as binding pockets and interactions between anesthetics and cytoplasmic proteins. Previous studies demonstrated the binding of VAs to tubulin. Since actin is the other major component of the cytoskeleton, this study involves an investigation of its interactions with four major anesthetics: halothane, isoflurane, sevoflurane, and desflurane. Molecular docking was implemented using the Molecular Operating Environment (MOE) software (version 2022.02) and applied to a G-actin monomer, extrapolating the relative binding affinities and root-mean-square deviation (RMSD) values. A comparison with the F-actin was also made to assess if the generally accepted idea about the enhanced F-to-G-actin transformation during anesthesia is warranted. Overall, our results confirm the solvent-like behavior of anesthetics, as evidenced by Van der Waals interactions as well as the relevant hydrogen bonds formed in the case of isoflurane and sevoflurane. Also, a comparison of the interactions of anesthetics with tubulin was made. Finally, the short- and long-term effects of anesthetics are discussed for their possible impact on the occurrence of mental disorders.

## 1. Introduction

Modern medicine has significantly improved clinical outcomes owing to the use of general anesthetics, a unique drug category that enables painless surgical procedures and life-saving treatments. Anesthetics, as a result of their amnesic and analgesic properties, can be used to induce unconsciousness, reduce pain, and establish a sedation state in patients undergoing medical treatments. The possibilities and success of surgical treatments have significantly increased due to their use such that surgeons can perform complex procedures that would have been impossible otherwise due to the excruciating pain they would have caused [1]. Anesthetics can be used in a variety of ways: For instance, inhalation anesthetics, which are the subject of this study, are gases or volatile liquids that the patient inhales after being vaporized, whereas intravenous anesthetics are delivered directly into the bloodstream, quickly inducing unconsciousness and maintaining it throughout the treatment. In spite of over a century of research in this field, the exact molecular mechanism of action of VAs is still unclear. However, we do know that they bind to several target proteins [2], especially to those belonging to the nerve cell membrane; these protein targets are believed to differ between intravenous and volatile anesthetics [2,3].

Intravenous anesthetics (IAs) induce anesthesia by reaching the central nervous system (CNS) through the bloodstream and achieving high concentrations in the CNS in a very short time. The most commonly used IAs are propofol, ketamine, thiopental, etomidate, and midazolam, which are small lipophilic molecules characterized by a short-acting effect, rapid metabolism, and excretion. The first three cited are reported to be the most potent and efficacious in medicine. Consequently, the patient can benefit from a rapid recovery after the surgical procedure. The main side effects of the use of general anesthetics are temporary confusion, nausea, and shivering [4]. While these drugs are considered very safe, depending on the surgical procedure, some patients having particular conditions are more at risk of negative outcomes such as heart attack, stroke, and pneumonia.

It has been known for many decades that anesthetic action is related to a drug’s hydrophobicity, its permanent dipole, and polarizability. It is generally accepted that anesthetics bind to nonpolar regions within brain proteins [5]. IAs’ lipophilicity is essential for the rapid crossing of the blood–brain barrier and rapid onset of action; this explains why these drugs act quickly. Although it is well established that primary sites of anesthetic mechanisms are a variety of membrane-bound proteins and ion channels, anesthetic binding to tubulin, the protein subunit of cytoskeletal microtubules, has also been implicated in several studies [6,7].

Since general anesthetics appear to affect the cytoskeleton reorganization by binding to tubulin [7], it is logical to analyze if cytoskeletal actin can be a potential site for binding of IAs. Actin is the primary determinant of cell morphology, motility, and vesicle trafficking, so it offers a putative site of action for IAs and can play a role in this process.

Despite their enormous benefits, anesthetics are still the subject of several concerns and open issues. The possibility of harmful impacts on essential physiological processes is one of the main worries. Both the administration and recovery phases of anesthesia require careful monitoring and management due to the potential dysfunctions that might cause cardiovascular instability, respiratory malfunction, changes in thermoregulation, and neuronal activity. Moreover, underlying medical disorders, age, and concurrent drug use are just a few examples of individual patient characteristics that can affect how they respond to anesthesia and raise the risk of complications [8,9]. One of the most frequent conditions experienced in the postoperative period is cognitive activity impairment, followed by delirium, memory loss, and impairment in executive functions [10]. Importantly, anesthesia together with surgical procedures seem to accelerate the progression of neurodegenerative pathologies such as Alzheimer’s disease and Parkison’s disease [11], as well as other neuronal disorders such as schizophrenia and autism [12,13]. For this reason, unraveling the complexity of anesthesia is crucial to significantly improving patient care and to fully understand the mechanisms through which anesthetics interact with certain target receptors such as gamma-aminobutyric acid (GABA) or N-methyl-D-aspartate (NMDA) receptors [14,15], as well as cytoskeletal proteins such as tubulin and actin [16].

The first accepted hypothesis about the molecular mechanism of volatile anesthetics follows the Meyer–Overton correlation regarding anesthetics dissolving in the lipid plasma membrane of neurons, modifying their mechanical properties to block action potential transmission [14]. The major lipidic components present in the brain are sphingolipids and cholesterol, which are located in specific macrodomains of membrane rafts [17], and they are crucial for enhancing motility in neuron growth and signaling [18]. However, regarding the possible effects on such changes in membranes, it can be stated that, at common anesthetic doses used in medical treatments, no substantial modifications occur, even at concentrations ten times higher in the case of nitrous oxide, halothane, and cyclopropane [19]. This is in line with a study showing very little dependence of the bilayer/gas partition coefficient on the lipidic bilayer content in the case of hydrocarbon anesthetics [20]. A new interpretation of the Meyer–Overton rule has been promoted by Cantor, suggesting a change in the lateral pressure profile of the lipid bilayer, favoring an increase in the fluidity and a variation in the thickness of such [21]. Computational results obtained by Zizzi et al. [22] demonstrate a direct relation between the bilayer bending modulus and the partition coefficient. This evidence suggests a new point of view regarding the interaction between the lipid membrane and anesthetics, favoring possible channel protein modifications with consequential effects on the cytoskeleton and the related embedded proteins.

Subsequent research has supported a second hypothesis according to which these chemicals bind to and influence a variety of neuronal proteins, ion channels, and neurotransmitter receptors [23,24,25]. In this regard, Eckenhoff et al. [26,27] provided some structural requirements about the specific and nonspecific binding of different classes of anesthetics to proteins in an aqueous-phase interaction, highlighting a direct relation with the supersecondary structure of proteins and the apposition of 3–4 helices, creating a suitable hydrophobic domain. The so-called ‘allosteric competition’ suggests interactions between multiple binding sites at the lipid–protein/protein–protein interfaces, influencing both protein dynamics and conformational changes, as well as the capability to donate hydrogen atoms [27]. Considering this connection, it is crucial to report some experimental results obtained by Eckenhoff et al. [28]. Halothane was found to bind to bovine serum albumin (BSA), with a ∆G° ≈ −3.7 kcal/mol (25 °C), as well as isoflurane and methoxyflurane, with ∆G° ≈ −4.3 kcal/mol (22 °C) and ∆G° ≈ −4.6 kcal/mol (22 °C), respectively. These results were also validated by Dubois et al. [29], who reported competitive binding to the BSA of these anesthetics, specifically sevoflurane, with ∆G° ≈ −4.9 kcal/mol (22 °C), with an emphasis on target protein conformations [28,30]. Sevoflurane is strictly correlated with folding, which seems to be favored in the case of mammalian β-barrel proteins, and in another study considering halothane and isoflurane as the subjects of investigation, ∆G° values are −5.5 and −5.4 kcal/mol, respectively [31].

Regarding the aforementioned involvement of ion channels, notably, a binding pocket at the interface of TM2, TM3, and TM4 helices of TREK1 has been identified with site-specific interactions of isoflurane [32,33], as well as Kv1.2 modulation by sevoflurane [33,34]. Isoflurane seems to also directly interact with the voltage-gated sodium channel NaChBac at sites located in the S4-S5 linker and at the extracellular surface [35]. A similar result was found for propofol, but it caused the inhibition of such a channel, interacting with common interfaces but at different binding sites compared to isoflurane [36].

GABA_A_ receptors [37] are well-documented targets not only for the binding of isoflurane and sevoflurane, which share a binding site located within the β+/α− interfaces of the α1β3γ2L GABAA receptor, but also at different binding sites, as is the case for the potassium channel [32,34]. Also, propofol modulates GABA_A_ receptor content, with a direct effect on the cytoskeleton, enhancing actin polymerization, due to an increase in intracellular calcium [38]. This result is particularly considerable, knowing the modulation of ion channel anchoring and, in general, the activity of the cytoskeleton involving both actin and tubulin [39]. These two proteins engage in crosstalk during neuronal growth and cooperate in polymerization, promoting such growth with the mediation of several proteins such as tau or motor proteins such that F-actin impacts the microtubule dynamics [40]. For this reason, the localization of actin in dendritic spines inside neurons has been hypothesized to be implicated in the postsynaptic action of VAs at excitatory synapses in the brain [41,42]. Moreover, actin has been linked to anesthesia-related processes such as altered synapse function and neural plasticity, but the exact mechanism of how anesthetics and actin interact is still not entirely clear [43].

The present study is focused on exploring the interactions between volatile anesthetics and actin. By creating a detailed 3D model of actin using crystallographic data and employing molecular docking techniques, we aim to investigate the binding modes and binding affinity of several important VAs (isoflurane, desflurane, sevoflurane, and halothane) toward actin. This research aims to shed light on these interactions and their potential effects on actin dynamics, cellular processes, and signaling pathways. Also, considering the close associations between actin and microtubules [40], our findings are compared to a previous study of the interaction between anesthetics and tubulin [7]. Hence, we can gain a better understanding of how anesthetics interact with different cytoplasmic proteins. 

## 2. Results

### 2.1. Actin

Actin is an important protein present in all eukaryotic cells, accounting for 15–20% of the total protein mass. It is an essential part of the cytoskeleton, which is a dynamic network of filaments that supplies structural support and carries out various cellular functions such as motility and transport [44]. Actin microfilaments consist of two chains of globular subunits (G-actin) that spiral around each other, forming filaments (F-actin). Actin filaments are polar, with a positive (barbed), fast-growing end and a negative (pointed), slow-growing end. 

Six different actin isoforms are distinguished, two striated muscle α-skeletal and α-cardiac, two α-smooth muscle actins, and two β- and γ-cytoplasmic ones that are encoded by separate genes in vertebrates [45]. The two isoforms of major interest for the purpose of our study are the cytoplasmic β- and γ-actin, which are essential for cell survival. These two isoforms differ only by four amino acids located at positions 1, 2, 3, and 9. The selective control of cytoplasmic actin isoform expression leads to functional specialization: γ-actin is responsible for cellular plasticity and motility, whereas β-actin is responsible for contraction and intercellular adhesion [46]. 

In this study, we focus on β-actin since it is particularly important in the context of brain cells due to its function in neuronal growth, synaptic plasticity, and cell migration. Synaptic plasticity is a key mechanism that supports learning and memory processes, and it refers to the synapse’s ability to undergo activity-dependent changes in strength [42,43]. β-Actin helps to create and maintain the intricate cytoskeletal network within neurons, which is required for normal functioning. Furthermore, it plays a role in cell migration throughout the brain. Neurons must migrate to the right sites throughout neurodevelopment in order to build functioning circuits. Finally, it is important to underline the high concentration of actin filaments, especially β-actin, in the cell’s filopodia, which are thin protrusions on the surface of cells that play a role in cell movement and guiding. Hence, they are required for neuronal growth and axon guidance [47,48]. 

β-Actin consists of several functional domains that play specific roles in structure and interactions with other proteins or molecules [49]. For instance, the binding site of a globular β-actin monomer is the specific location of the protein where it may interact with other molecules or participate in actin filament formation. The generally accepted classification of the structure of β-actin consists of the following three subdomains:

- The first subdomain (SD1) is the N-terminal domain of β-actin. It may be involved in protein interactions and actin filament assembly regulation. This region contains residues 4–39.
- The second subdomain (SD2), the central one, is involved in interactions with actin-binding proteins (ABPs) and other actin monomers to produce filaments. This core region includes residues 40–164.
- The third subdomain (SD3), the C-terminal domain, contains the amino acids found at the C-terminus of β-actin. This domain may interact with other proteins or molecules, regulate actin activities, and take part in many cell signaling cascades. This region comprises residues 165–374.
- Actin polymerization aids in the internalization of membrane vesicles, which helps regulate the composition of the cell membrane and the cell’s interaction with the environment. According to some research, volatile anesthetics can alter cytoplasmic actin polymerization through a variety of methods, including actin filament stability, the suppression of actin polymerization, and the regulation of actin filament activity. A broad variety of actin-binding proteins tightly regulate the polymerization and depolymerization of actin filaments, allowing for the dynamic remodeling of the cytoskeleton in response to cellular demands. Actin has been linked to a variety of pathological illnesses, making it an appealing therapeutic target.

### 2.2. Anesthetics

Volatile anesthetics (VAs) are a class of drugs commonly administered via inhalation through a mask or an endotracheal tube. At the macroscopic level, the general physiological effects are decreased blood pressure and reduced cerebral metabolism. Their potency can be easily adjusted by varying the dosage of the inhaled gas mixture. Different concentrations can cause respiratory depression, low blood pressure, and reduced cardiac function [50]. At the microscopic level, these anesthetics act on cells by interfering with neurotransmitter receptors, and they interact with the phospholipidic membrane, increasing fluidity and ion channel activity. Furthermore, they affect the activity of neurotransmitters in the brain, particularly gamma-aminobutyric acid (GABA) receptors, enhancing their inhibitory action and leading to the suppression of neuronal activity and the induction of anesthesia [14,15].

Regarding the specific interactions with actin, VAs can alter its polymerization and play a role in depolymerization and the overall stability of actin filaments. VAs can also affect the function and interactions of actin-binding proteins (ABPs), which regulate actin dynamics [51]. Below, we briefly discuss the anesthetic molecules investigated in this study.

Halothane (2-bromo-2-chloro-1,1,1-trifluoroethane or F_3_CCHBrCl) belongs to the class of halogenated hydrocarbons. It is barely soluble in water but highly soluble in organic solvents such as alcohol, chloroform, and ether. It has a molecular weight of 197.38 g/mol, and its boiling point is 50.2 °C. Its immobilizing effects have been attributed to its binding to potassium channels in cholinergic neurons. Halothane’s effect is also likely because of binding to NMDA and calcium channels, causing hyperpolarization. This anesthetic has been widely used in the past but is now less common due to some concerns about side effects such as hepatotoxicity [52]. 

Other VAs of interest in this study are isoflurane (2-chloro-2-(difluoromethoxy)-1,1,1-trifluoroethane or C_3_H_2_ClF_5_O), which has been used in clinical practice for many years, and sevoflurane (1,1,1,3,3,3-hexafluoro-2-(fluoromethoxy)propane or C_4_H_3_F_7_O). Isoflurane is a halogenated hydrocarbon compound. It is a clear and colorless liquid at room temperature; its molecular weight is 184.49 g/mol, and it boils at approximately 48.5 °C [53]. Sevoflurane belongs to the class of dialkyl ethers; it has a pleasant odor, and it does not damage the airway. It has a molecular weight of 200.05 g/mol, and its boiling point is 58.8 °C. In clinical environments, both isoflurane and sevoflurane have a rapid onset and offset action; they offer good muscle relaxation and analgesic properties, which are beneficial during surgical procedures. The low solubility in the blood that characterizes them results in rapid equilibration between the alveolar gas and the brain, facilitating rapid induction and emergence from anesthesia, which makes them suitable for both short and long surgical procedures [54,55].

Finally, desflurane (2-(difluoromethoxy)-1,1,1,2-tetrafluoroethane or C_3_H_2_F_6_O) is an aliphatic acyclic compound, which has low solubility in water but dissolves easily in organic solvents such as alcohol and ether. It weighs 168.04 g/mol and boils at 22.8 °C [56]. Unlike other volatile anesthetics, it is particularly suitable for short procedures. However, the specific characteristics of desflurane may differ from those of halothane and other volatile anesthetics. For example, desflurane has been reported to produce more tachycardia and hypertension on induction than isoflurane [57,58].

Regarding the specific activity on actin filaments, there is no detailed information about direct interactions in the cytoplasmic membrane, but it is shown that anesthetics mediate the reversible F-to-G-actin transformation inside the cell by interacting with some actin-binding proteins (ABPs) such as cofilin, drebrin, and CamKII, which are also known to regulate dendritic protrusions’ formation and synaptic plasticity [51].

In light of the VA–actin analysis, it is crucial to refer to the Meyer–Overton theory that, despite several deficiencies, is currently the accepted theory explaining the mechanism of action of these kinds of compounds [52]. To be more precise, it is stated that anesthetic potency is correlated with their solubility in lipids. Based on a thermodynamic process involving anesthetics, there exists a high degree of correlation between the oil–water diffusion coefficient and fatty membranes possibly due to physical effects, metabolic variations, or intrinsic characteristics of anesthetics. In general, anesthetic potency is directly proportional to their solubility in liquids, and for this reason, research has focused on hydrophobic cores as potential binding sites for anesthetics [59,60]. It is essential to clarify that this is a simplified model that does not account for other several factors that characterize anesthesia. Indeed, additional parameters and more complex models need to be considered when researching this topic. Therefore, the current study is focused on the investigation of hydrophobic cores of the β–G-actin with respect to the generally accepted division of actin into three different subdomains and on using molecular docking to gain fresh insights into the interaction of these volatile anesthetics at an atomic scale to evaluate their influence on the cytoskeleton network. We first consider actins and then a comparison is made to tubulins, referring to an earlier study [7].

### 2.3. Data Analysis

The binding affinity found for each anesthetic is reported as the mean value ± the standard deviation (Table 1); the same is also the case for the RMSD value (Table 2).

Sevoflurane had the highest negative value of binding energy: −3.48 ± 0.15. However, there was no significant difference among the anesthetics analyzed.

**Figure 1:**
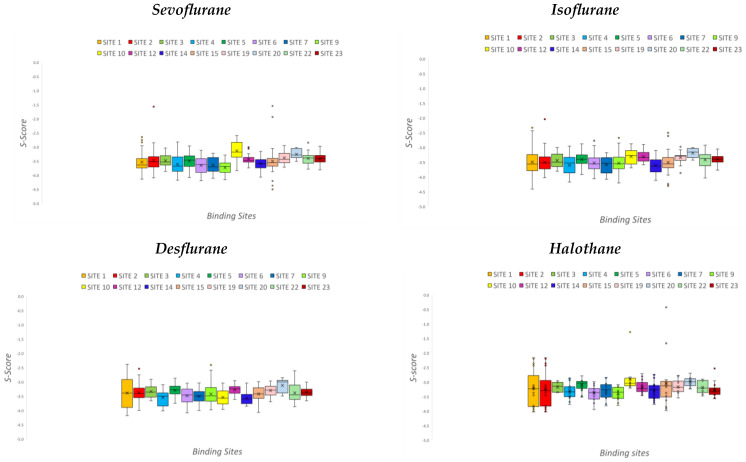
Boxplots representing S-score values across all poses for each anesthetic.

Such data have been also represented with boxplots to highlight the differences regarding both the binding pose and the binding site (Figure 1).

**Figure 2:**
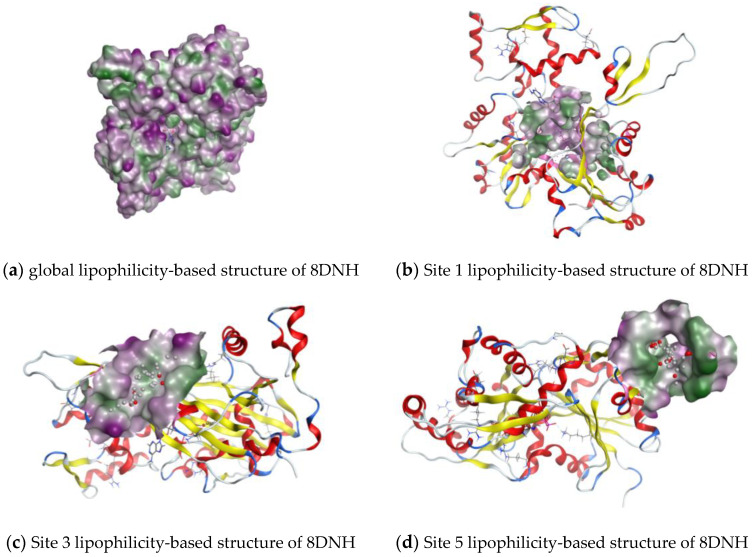

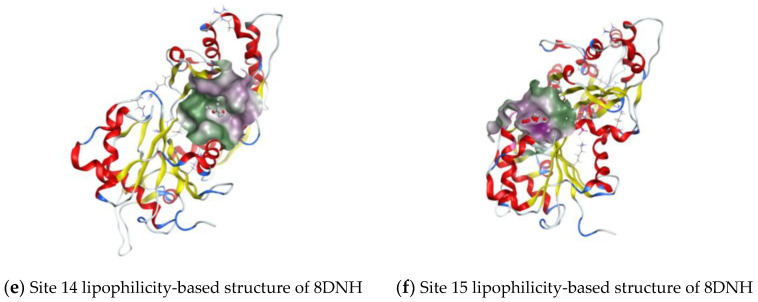
Lipophilicity-based surfaces. The color code used is as follows: Alpha Helix—red ribbon, Beta sheet—yellow ribbon, Coils or Loops—blue and white ribbon, Lipophilicity—green, Hydrophilicity—purple.

The images related to the qualitative analysis are reported below (Figure 2). The surfaces were generated using the MOE tool to visualize the lipophilic regions of the protein and the pockets. 

**Figure 3:**
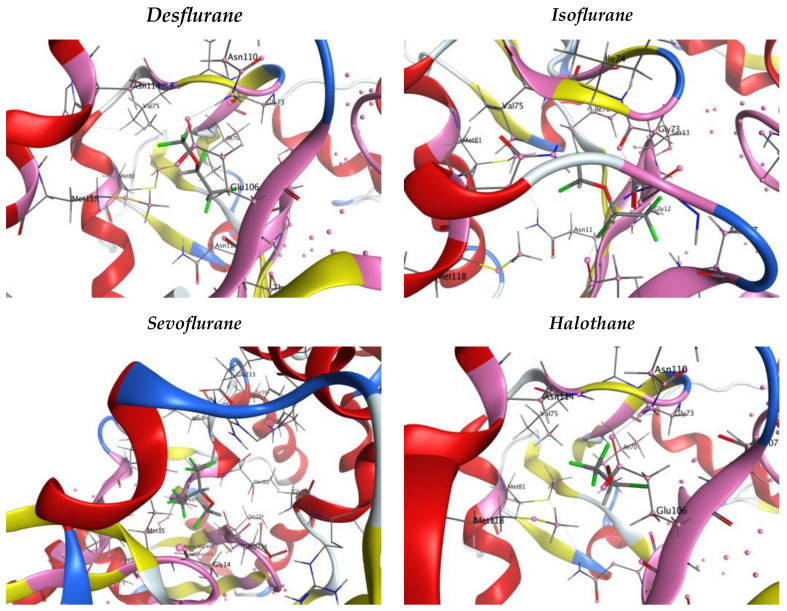
The 8DNH snapshots of Site 1 binding pockets and VAs. The color code here is as follows: Alpha Helix—red ribbon, Beta sheet—yellow ribbon, Coils or Loops—blue and white ribbon, Contact atoms of the selected binding pocket– pink ribbon and atoms, Carbon backbone—grey bars, Nitrogen atoms—blue bars, Oxygens—red bars, Fluorines—green bars.

To have a quantitative measure of the hydrophobicity of each site, the ‘hydrophobic’ residues were counted, as shown in Table 3. Also, four snapshots of the analyzed VAs inside the largest binding pocket are presented to better visualize the interplay between hydrophobic, hydrophilic residues, and VAs (Figure 3). The choice of reporting only the first site is due to compactness and also because Site 1 is one of the most hydrophobic, as reported in Table 3.

**Figure 4:**
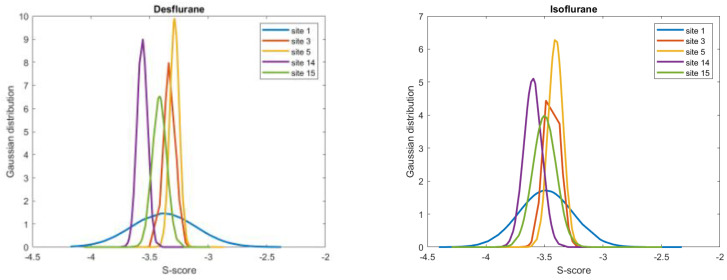

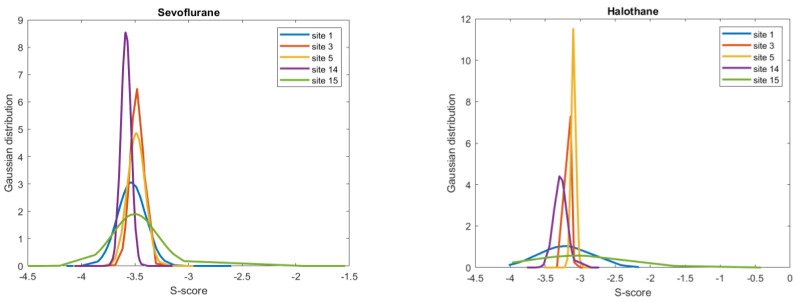
Gaussian distributions of the S-score values for the selected sites.

Furthermore, the Gaussian distribution is reported for the five selected binding sites (Figure 4). Each distribution is centered around the middle interval of the entire range, including the one for Site 1, which has a larger standard deviation than the others.

Regarding the RMSD value, sevoflurane had the highest value, but it differed by 0.28 from halothane. Indeed, halothane’s RMSD was 160.59 ± 12.68, whereas sevoflurane’s RMSD was 160.62 ± 13.43 (Table 2).

A custom-made MATLAB code was appositely developed to differentiate the sites depending on their location with respect to the division in subdomains, and the ones with a percentage of belonging greater than 50% were selected. To be more specific, the following sites were finally considered for the investigation and representation of the results:

- SITE 1: 53%;
- SITE 3: 100%;
- SITE 5: 77%;
- SITE 14: 75%;
- SITE 15: 100%.

A final visual inspection of both F- and G-actin was also carried out with a quantitative analysis in terms of putative binding sites that were still present when the G-to-F-actin polymerization occurred. The monomer appeared to be almost completely superimposable in both cases. Regarding the availability of the sites, in most cases, the predicted binding sites for 8DNH (G-actin) were still available in F-actin even if not entirely in terms of identical residues. To be more precise, 20 sites were extrapolated using the Site-Finder applied on the superposition of the 8NDH with a single chain of 8D17: Eight sites disappeared, while the others were embedded within larger sites in F-actin. When considering the difference between the Site-Finder applied to the 8DNH used in this study and the Site-Finder applied to the whole structure of 8D17, 5 out of 97 sites were not present. 

## 3. Discussion

The issue of the specific mechanisms of action of anesthetics is still unresolved. The Meyer–Overton theory provides a link regarding the possible interactions between anesthetics and the lipid membrane bilayer that supports the hydrophobic profile of the anesthetic binding sites based on a thermodynamic action when anesthesia is induced [52]. Recent studies also support a more specific anesthetic–protein interaction instead of any modification of the lipid membrane [23,24,25].

In order to improve our understanding of anesthetic mode(s) of action, molecular docking was performed by employing four different anesthetics and the actin protein, in its globular form. An analysis was performed in parallel focusing on the nature of the predicted binding sites using MOE’s Site-Finder module and the ligand interaction results obtained from the docking of the best poses selected for each anesthetic and the β-globular actin. In general, it was based on the hydrophobic profile of the binding sites, as discussed above [59,60]. Regarding their location with respect to the monomer of actin, the first site was the largest, and it was located rather internally in the structure but it also generated enough volume at the oligomeric interface [11], whereas the others were more external. The first criterion (lipophilic surfaces and the quantification of hydrophobic residues) confirmed the general idea of the interaction in the hydrophobic cores of the main protein. Indeed, the selected sites appeared to be the most hydrophobic ones. However, no significant differences for these sites were observed in terms of the S-score. 

It is noteworthy that the total number of poses extrapolated from the software was lower than that of the refinement set before performing the docking. This result was interpreted as a consequence of the size of the anesthetics relative to the size of the protein binding pockets. Their solvent-like behavior and the presence/absence of rotatable bonds may lead to a higher number of poses. For instance, halothane had no rotatable bonds, and this resulted in having the lowest maximum number of poses (24) versus the others, whose maximum number was around 80. In particular, the maximum number of poses in desflurane was 88 and that of isoflurane was 86, both having three rotatable bonds, whereas the maximum number of poses in sevoflurane was 89, with four rotatable bonds. 

Investigating more closely the ligand interaction results obtained from the docking of the best pose selected for each anesthetic and the β-globular actin, we were able to confirm a solvent-like behavior in the majority of the cases. Indeed, considerable interactions were mostly due to Van der Waals forces during the reaction for the case of halothane and desflurane, while only in a few cases, some hydrogen bonds were formed. In the case of isoflurane and sevoflurane, a more remarkable result in terms of hydrogen bonds was obtained because, in the former, 11 out of 16 sites were involved in such bonds formed with actin residues; in the latter, 9 out of the 16 sites were involved. This finding may support the idea of other emerging models that hypothesize a hydrophilic characteristic of the pockets involved in the interactions together with the direct interaction of anesthetics with proteins [14]. This intriguing interplay between Van der Waals interactions and hydrogen bonds can lead to more stable anesthetic–actin complexes, even if reversible in most cases, which can possibly favor β-amyloid oligomerization, leading to a toxic final quantity that may accelerate neurodegenerative pathologies such as Alzheimer’s disease [11,61]. 

However, it should be stated that the particular location of each binding site is strategic for the consequent effects on the entire protein. Indeed, there could be an opposite effect of anesthetics depending on the binding pocket characteristics. For instance, the availability of intermonomer binding sites allows for the formation of a hydrophobic cleft at the interface between two actin monomers so that they disassemble by means of anesthetics. On the other hand, more external pockets, between SD1 and SD3, may modulate or prevent the D-loop formation, which is another hydrophobic cleft involved in bonds with ATP or other nucleotides [49,60]. 

Examining specifically the S-score, the most negative score was found for sevoflurane, indicating its highest affinity for tubulin [7]. This result is not unexpected since it is the largest molecule in terms of molecular weight and thus has a tighter interaction with the monomer. Second in weight is halothane, which yielded the lowest binding energy value but the highest RMSD value. For instance, as previously mentioned, sevoflurane has four rotatable bonds versus halothane, which has zero. Such a result in terms of the RMSD value may seem quite unexpected, but it has to be taken into consideration that the algorithm from which the results are extrapolated corresponds to the random poses tested. Moreover, halothane is not a hydrogen bond donor, and this may explain why it has the highest RMSD, i.e., as a result of weak interactions due to weak hydrogen bonding. A general note about the standard deviation of the RMSD among the different poses analyzed is that there was no substantial difference among all the sites for the four anesthetics. The poses were not very dissimilar from one another, and no preference was found. 

To proceed with a more detailed characterization of the binding sites provided by the MOE Site-Finder, we investigated the general assumption regarding the subdomains of the actin protein. We examined a specific interaction of anesthetics with actin-binding proteins (ABPs) that caused an F-to-G-actin transformation more than the actin itself, resulting in the disruption of neuronal filaments and thus inducing synapsis [62,63]. We found an association between the most hydrophobic sites and their location with respect to the actin SD2, and it could be interpreted that such sites are potential target pockets for the interaction. Furthermore, they promote a direct anesthetic–protein interaction, while ABPs have an indirect effect on other proteins since actin favors structural changes in the cytoskeleton associated with synaptic plasticity. 

For a comparison between our results regarding actin and anesthetic interactions with tubulin [7], halothane and desflurane were both considered in the analysis. However, no significant difference was found between the binding affinities in terms of the order of magnitude. Numerically, in the case of the interactions with tubulin, the binding energies were slightly more negative than those for the actin monomer. As a general consideration, also involving ethylene and methoxyflurane, the range of S-score variation was comparable. However, it should be emphasized that the data were extrapolated by using two different software packages with different environmental conditions and algorithms implemented for the purpose. 

For a comparison between F-actin and G-actin, it is crucial to clarify the different source organisms from which both 8DNH and 8D17 are derived. This is an essential aspect that may lead to structural variations in the protein, thus resulting in modifications of their functionality and/or structure within the organism so that various effects might be generated. 

The sites extrapolated from the MOE Site-Finder, which was applied to 8DNH superimposed on a single chain of 8D17, yielded 20 available sites with respect to the 23 originally selected for the single monomer of 8DNH, 8 of whom were not present in the superposition. A similar condition was observed for the case of the comparison between a single monomer of globular actin and a complete filamentous actin structure, where 5 sites out of the 97 sites were not present. Since this was a qualitative analysis, it might be assumed that most of them were conserved and still present after the G-to-F-transformation. As an additional note, these 8 specific binding sites, which were not available, similar to the second case in which 5 out of the 97 sites were not present, were divided into pairs of residues and subsumed by larger but differently located ones. With respect to the molecular pathways involved, the identified binding sites should be connected with specific protein interactions with actin, which would provide us with a more comprehensive analysis of how anesthetic molecules affect the network of cytoskeletal proteins via weak but numerous interactions in the neuron [60]. The potential physiological consequences of these compounds, i.e., not only the short-term effects but also mostly the long-term effects on the CNS, are not negligible. The disassembly of the F-actin has a direct impact on neurite elongations even at nontoxic concentrations with respect to the commonly used anesthetics [59]. Additionally, any external effects on actin remodeling disturb the cytoskeleton, which may even lead to mutations in synapsis with an impact on the development of several mental disorders [13]. 

## 4. Material and Methods

As the first step in the workflow, a structure related to globular actin was chosen from the Protein Data Bank (PDB) and was modified properly. This was imported to the MOE [64], the software used to carry out molecular docking simulation, and then using the MOE Site-Finder, the binding sites of the selected structure were identified. Finally, the structures of the volatile anesthetics were imported from the PubChem database, and molecular docking was performed. As a final step, the results obtained from docking were analyzed and compared with those of a previous study on tubulin [7].

The globular actin model was downloaded from the Protein Data Bank (PDB): Its reference number is 8DNH. The choice of such a model was made by examining the state of the art in the field, as presented in the tables below (Table 4 and Table 5), including both the cytoplasmic β- and γ-actins. The following features were considered: the resolution of the model, the technique used to derive it, its recentness, and the chain’s completeness in terms of eventual missing residuals. The source organism is common for each of the models, namely *Homo Sapiens*. The chosen model corresponds to the Cryo-EM structure of nonmuscle beta-actin [65]. It was uploaded on 12 April 2023 to PDB; electron microscopy was used with a final resolution of 2.99 Å. The reported coverage was 100% for chain 2–375: the first residue was added with the help of the MOE Sequence Editor, and residue 73, reported to be modified, was restored to its correct form.

Furthermore, the ‘Quick Prep’ command was used, along with ‘Protonate 3D’, to perform some adjustments following the MOE algorithm, with default settings, through which the protein was restructured in terms of charge, energy conformation, end loops, and global structure refinements. Regarding the examined anesthetics, each model was downloaded from PubChem. The reference numbers are halothane (CID—3562), isoflurane (CID—3763), desflurane (CID—42,113), and sevoflurane (CID—5206). Then, they were uploaded to the MOE to perform docking. This procedure allowed us to analyze the possible interactions between each ligand and the receptor in different binding sites.

The command ‘Site-Finder’ was used in order to identify putative binding sites, which resulted in 23 suitable sites. First, these sites included the ones with a positive reported PLB value. In addition, the lipophilicity investigation involved those with a negative reported PLB value. To this end, a surface for each binding pocket was generated to provide a qualitative measurement of the lipophilicity of each site so that the most hydrophobic ones were selected. In total, 16 sites were retained for further analysis.

The Dock Algorithm within the MOE was implemented in a seven-step process, setting the following parameters for each anesthetic: The pose placement was set to 200, and refinement was set to 100. A final table for each site and each anesthetic was created (for a total of 60), containing the following features: pose, S-score, and RMSD. The best pose was selected by taking into account the conformation with which the highest negative S-score value was associated. Each of such poses was sent to the MOE for the visualization of the ligand interactions. Furthermore, for more consistent discrimination among the several identified binding sites, a MATLAB custom-made code was developed to better inspect them according to the percentage of belonging to the reported actin domains, bringing into focus the second domain (SD2), due to its known involvement with interactions with ABPs. The criterion chosen to select sites regarding the percentage of belonging to SD2 was as follows: Among all sites, those having a percentage of belonging greater than 50% were considered. As a result, the sites identified this way were the first, the third, the fifth, the fourteenth, and the fifteenth from the Site-Finder.

The mean values of the S-score are reported in two different representations, namely a global one (Table 1) and boxplots (Figure 1), to better visualize the variations among all the sites for each anesthetic. In addition, a Gaussian distribution was determined for the five sites with respect to the criteria previously mentioned (Figure 4). With regard to hydrophobicity, a quantitative analysis was performed by calculating the number of residues considered particularly hydrophobic. The literature provides discordant suggestions about which residues to consider because discrimination refers to different characteristics. However, the residues considered for the analysis were leucine (LEU), isoleucine (ILE), valine (VAL), phenylalanine (PHE), methionine (MET), and proline (PRO) [66,67]. Data are reported in Table 3, together with images of the surfaces of the five selected sites; one image illustrates the whole structure, created on the basis of lipophilicity (Figure 2). A final qualitative analysis provided a comparison between G- and F-actin. In particular, the 8DNH model was visually compared to a model of filamentous actin. This model was downloaded from PDB (ID: 8D17). It is a sample of straight F-actin 1, an ADP nucleotide state, and the source organism is *Gallus gallus* [68]. It was reconstructed using electron microscopy, with a final resolution of 3.69 Å. The comparison consisted of analyzing and checking if the available binding sites of the F-actin (8D17) were equal to the ones found by applying the Site-Finder to G-actin (8DNH). In particular, Site-Finder was evaluated both globally on 8D17 and by superposing the 8DNH model. The superposition was computed after the pre-alignment of a single chain of the F-actin (8D17) with the human G-actin (8DNH), using the MOE Sequence Editor tool. All the results are reported and discussed in this article.

## 5. Conclusions

The analysis of the obtained results shows that the investigated anesthetics exhibit pseudo-solvent behavior toward actins. The analyzed bunding poses were slightly different from each other but represented a similar overall conformation. Qualitatively, none was optimal, and they all showed comparable binding energy values; thus, no pose was found to be superior to others in terms of binding affinity for any site in any of the four anesthetics. However, these results contribute to the body of evidence regarding specific interactions between proteins and anesthetics. Indeed, they are generally aligned with the experimental results by Eckenhoff et al. [26,28,30,31,32,34,35,36,37] and the computational work of Zizzi et al. [7]. They shed light on the possible mechanisms of action of anesthetics with similar general characteristics but specific differences depending on the chemical properties of the anesthetic molecules investigated. As an example, we found opposite effects of halothane [59] and propofol [38] on actin, with the former favoring the F-actin disassembly, while the latter promoting its assembly. For this reason, a detailed analysis is needed about the related effects of these processes on neuronal growth or degradation. On the other hand, a common pattern exists when correlating anesthetic binding with specific motifs in the tertiary and quaternary structure of proteins [27]. 

It must be kept in mind that molecular docking alone is not sufficient to address the complex issue of a drug’s mode of action, as several other effects such as solubility, permeability, and off-target interactions play major roles, in addition to the composition of the medium in which these compounds interact with proteins and membranes. Studying at a static level the interaction between volatile anesthetics, dynamic fluids, and the actin protein is therefore necessary. For example, pH and ionic concentration changes may cause conformational changes in G-actin, which might destabilize F-actin and hence modify the action of anesthetics [69,70]. This can be investigated using molecular dynamics, as shown by Zizzi et al. [7]. Including molecular dynamics analysis in future work would make it possible to observe the effects of the environment on the anesthetic action over time. Finally, it is essential not to underestimate the potency–toxicity issue when considering anesthetics’ dosage because this affects the CNS in an irreversible way. In this regard, melatonin may help in reducing the quantity of anesthetic needed for sedation, especially if used as a pretreatment before surgical procedures, and, due to its analgesic and antioxidative properties, it may also safeguard the cytoskeleton structure, i.e., the microtubules and microfilaments forming neurons. In this way, neuronal loss and the related impairments in cognitive functions and memory can be limited. Hence, additional computational and experimental studies on the adjuvant role of melatonin may result in improved administration of anesthetics with a reduction in potential neurological damage [71,72,73].

## Figures and Tables

**Table 1:** Mean value ± standard deviation of binding affinity.

Anesthetic	Binding Affinity (kcal/mol)
Desflurane	−3.39 ± 0.12
Halothane	−3.19 ± 0.14
Isoflurane	−3.44 ± 0.12
Sevoflurane	−3.48 ± 0.15

**Table 2:** Mean value ± standard deviation of RMSD.

Anesthetic	RMSD Value
Desflurane	159.53 ± 13.10
Halothane	160.59 ± 12.68
Isoflurane	124.30 ± 12.29
Sevoflurane	160.62 ± 13.03

**Table 3:** Hydrophobic residues for each pose.

Site	PLB	Hydrophobic Residues	Number of Hydrophobic Residues
1	4.55	VAL9 MET15 ILE70 VAL75 MET81 LEU104 MET118 VAL158 VAL338	9
2	0.25	PRO108 LEU109 VAL133 ILE135 PRO171 ILE174 VAL369 PHE374	8
3	0.21	VAL53	1
4	0.12	MET0 ILE4 PHE20 LEU348	4
5	0.11	PRO37 VAL42 MET43 MET46 ILE63	5
6	0.05	VAL133 ILE135 VAL138 LEU139 ILE164 LEU345 PHE351 MET354 PHE374	9
7	0.03	ILE340 ILE344	2
8	−0.02	MET189 LEU192 PHE199	3
9	−0.03	MET15 LEU215 MET304 PRO306	4
10	−0.18	VAL297 PRO331 ILE340	3
11	−0.19	ILE70	1
12	−0.29	PHE199 ILE207 LEU241 PRO242	4
13	−0.29	LEU66 PRO69	2
14	−0.35	VAL138 LEU141 ILE164	3
15	−0.37	PRO108 LEU109 PRO111 ILE135	4
16	−0.38	LEU235	1
17	−0.39	-	0
18	−0.39	LEU220 PHE222	2
19	−0.41	LEU175 LEU177	2
20	−0.41	PRO26 ILE340	2
21	−0.42	ILE63 LEU64	2
22	−0.56	VAL286 ILE288 MET324	3
23	−0.64	ILE4 PRO101 PRO129	3

**Table 4:** Cytoplasmic actin 1 (β-actin).

Code	Method	Resolution	Upload Date	Sequence Length	Missing Residues	Modified Residues	Related Study
6ICT	X-RAY DIFFRACTION	1.95 Å	FEB 2019	23—Chain E, G, H, I (66–88)	66, 86–88	73—HIC	Structure of SETD3 bound to SAH and methylated actin
7W28	X-RAY DIFFRACTION	1.79 Å	OCT 2022	16—Chain P (66–81)	-	73—N9P	Crystal Structure of SETD3-SAH in complex with betaA-4PyrAla73 peptide
6OX3	X-RAY DIFFRACTION	1.78 Å	AUG 2019	19—Chain E, G, H, I (66–84)	-	-	SETD3 in Complex with an Actin Peptide with His73 Replaced with Lysine
6ICV	X-RAY DIFFRACTION	2.15 Å	FEB 2019	23—Chain C, D (66–88)	66, 84–88	-	Structure of SETD3 bound to SAH and unmodified actin
6OX0	X-RAY DIFFRACTION	1.76 Å	AUG 2019	15—Chain Y, Z (66–80)	-	-	SETD3 in Complex with an Actin Peptide with Sinefungin Replacing SAH as Cofactor
6OX2	X-RAY DIFFRACTION	2.09 Å	AUG 2019	15—Chain Y, Z (66–80)	-	73—HIC	SETD3 in Complex with an Actin Peptide with the Target Histidine Fully Methylated
6OX1	X-RAY DIFFRACTION	1.95 Å	AUG 2019	15—Chain Y, Z (66–80)	-	73—HIC	SETD3 in Complex with an Actin Peptide with Target Histidine Partially Methylated
6OX5	X-RAY DIFFRACTION	2.1 Å	AUG 2019	18—Chain Y	-	-	SETD3 (N255A) mutant complexed with an actin peptide with His73 replaced by lysine
6MBK	X-RAY DIFFRACTION	1.69 Å	DEC 2018	15—Chains Y, Z	-	-	SETD3, a Histidine Methyltransferase, in Complex with an Actin Peptide and SAH, First P212121 Crystal Form
6MBJ	X-RAY DIFFRACTION	1.78 Å	DEC 2018	15—Chains Y, Z	-	-	SETD3, a Histidine Methyltransferase, in Complex with an Actin Peptide and SAH, P21 Crystal Form
6MBL	X-RAY DIFFRACTION	2.2 Å	DEC 2018	15—Chains Y, Z	-	-	SETD3, a Histidine Methyltransferase, in Complex with an Actin Peptide and SAH, Second P212121 Crystal Form
6OX4	X-RAY DIFFRACTION	2.29 Å	AUG 2019	15—Chains Y, Z	-	-	SETD3 (N255A) mutant in complex with an actin peptide
7W29	X-RAY DIFFRACTION	2.9 Å	OCT 2022	16—Chain P	-	73—ORN	SETD3-SAH crystal structure in complex with the peptide betaA-Orn73
3D2U	X-RAY DIFFRACTION	2.21 Å	JUL 2008	9—Chains C, G	-	-	Structure of UL18, a Peptide-Binding Viral MHC Mimic, Bound to a Host Inhibitory Receptor
6NBW	X-RAY DIFFRACTION	2.5 Å	GEN 2020	374—Chain A	41–47	73—HIC	Ternary complex of beta/gamma actin with profilin and AnCoA-NAA80
8DNH	E-MICROSCOPY	2.99 Å	APRIL 2023	375—Chain A, B, C, D	-	73—HIC	Non muscle beta actin
6LTJ	E-MICROSCOPY	3.7 Å	FEB 2020	375—Chain K	1, 13–16, 93–96, 373–375	-	Nucleosome-bound human BAF complex
7VDV	E-MICROSCOPY	3.4 Å	MAY 2022	375—Chain P	1, 13–16, 33–78, 93–96, 373–375	-	Human chromatin remodeling PBAF-nucleosome complex
7AS4	E-MICROSCOPY	4.13 Å	GEN 2021	374—Chain G	39–48	-	Recombinant human gTuRC
7P1H	E-MICROSCOPY	3.9 Å	NOV 2021	372—Chain B	38–44	70—HIC	Exo-Y-G-actin -profilin complex
6ANU	E-MICROSCOPY	7 Å	NOV 2017	375—Chain A, B, C, D, E, F	-	-	F-actin complexed with beta-III-spectrin-ABD
7QJ6	E-MICROSCOPY	7.8 Å	JAN 2022	374- Chain A	1, 40–49	-	Structure of recombinant human gamma-Tubulin Ring Complex 10-spoked assembly intermediate
7QJ9	E-MICROSCOPY	8.1 Å	JAN 2022	372—Chain E	1, 40–49	-	Structure of recombinant human gamma-Tubulin Ring Complex 10-spoked assembly intermediate
3J82	E-MICROSCOPY	7.7 Å	MAY 2015	374—Chain B, C, D	-	72- HIC	C-type lectin domain family 9 member A complexed with F-actin
3BYH	E-MICROSCOPY	12 Å	FEB 2008	374—Chain A	-	-	Actin-fimbrin ABD2 complex
3LUE	E-MICROSCOPY	15 Å	APR 2010	374- Chains A, B, C, D, E, F, G, H, I, J	-	-	Alpha-actinin CH1 model bound to F-actin

**Table 5:** Cytoplasmic actin 2 (γ-actin).

Code	Method	Resolution	Upload Date	Sequence Length	Missing Residues	Modified Residues	Related Study
6V63	X-RAY DIFFRACTION	2.02 Å	JAN 2020	23—Chain Y, Z (66–88)	85–88	-	SETD3 WT in Complex with an Actin Peptide with His73 Replaced with Glutamine
6WK1	X-RAY DIFFRACTION	1.89 Å	JUN 2020	23—Chain Y, Z (66–88)	85–88	-	SETD3 in Complex with an Actin Peptide with His73 Replaced with Methionine
6WK2	X-RAY DIFFRACTION	1.76 Å	JUN 2020	23—Chain C, Y (66–88)	85–88	-	SETD3 mutant (N255V) in Complex with an Actin Peptide with His73 Replaced with Methionine
6V62	X-RAY DIFFRACTION	2.36 Å	JAN 2020	23—Chain Y (66–88)	84–88	-	SETD3 double mutant (N255F/W273A) in Complex with an Actin Peptide with His73 Replaced with Lysine
7NVM	E-MICROSCOPY	3.1 Å	MAR 2022	375—Chain K	1–5, 35–49, 193–200, 231–260	-	Human TRiC complex in closed state with nanobody Nb18, actin and PhLP2A bound
8DNF	E-MICROSCOPY	3.38 Å	APR 2023	375—Chain A, B, C, D	-	73—HIC	Cryo-EM structure of nonmuscle gamma-actin
5JLH	E-MICROSCOPY	3.9 Å	JUN 2016	374—Chain A, B, C, D, E	-	-	Cryo-EM structure of a human cytoplasmic actomyosin complex at near-atomic resolution
6G2T	E-MICROSCOPY	9 Å	OTT 2018	375—Chain A, B, C, D, E, F	1–5	-	Human cardiac myosin binding protein C C1 Ig-domain bound to native cardiac thin filament
6CXJ	E-MICROSCOPY	11 Å	OTT 2018	375—Chain A, B, C, D, E	1–5	-	Cardiac thin filament decorated with C0C1 fragment of cardiac myosin binding protein C mode 2
6CXI	E- MICROSCOPY	11 Å	OTT 2018	375—Chain A, B, C, D, E	1–5	-	Cardiac thin filament decorated with C0C1 fragment of cardiac myosin binding protein C mode 1

## Data Availability

All data supporting the findings of the study are available from the corresponding author, J.A.T., upon reasonable request.
